# Maintenance of Miranda Localization in *Drosophila* Neuroblasts Involves Interaction with the Cognate mRNA

**DOI:** 10.1016/j.cub.2017.06.016

**Published:** 2017-07-24

**Authors:** Anne Ramat, Matthew Hannaford, Jens Januschke

**Affiliations:** 1 Cell and Developmental Biology, School of Life Sciences, University of Dundee, Dow Street, DD5 1EH Dundee, UK

**Keywords:** *Drosophila*, asymmetric cell division, mRNA localization, nanobody, neuroblasts, polarity

## Abstract

How cells position their proteins is a key problem in cell biology. Targeting mRNAs to distinct regions of the cytoplasm contributes to protein localization by providing local control over translation. Here, we reveal that an interdependence of a protein and cognate mRNA maintains asymmetric protein distribution in mitotic *Drosophila* neural stem cells. We tagged endogenous mRNA or protein products of the gene *miranda* that is required for fate determination with GFP. We find that the mRNA localizes like the protein it encodes in a basal crescent in mitosis. We then used GFP-specific nanobodies fused to localization domains to alter the subcellular distribution of the GFP-tagged mRNA or protein. Altering the localization of the mRNA resulted in mislocalization of the protein and vice versa. Protein localization defects caused by mislocalization of the cognate mRNA were rescued by introducing untagged mRNA coding for mutant non-localizable protein. Therefore, by combining the MS2 system and subcellular nanobody expression, we uncovered that maintenance of Mira asymmetric localization requires interaction with the cognate mRNA.

## Highlights

• Nanobody technology and the MS2 system can be used to alter mRNA localization
• *miranda* mRNA localizes in two distinct pools in *Drosophila* neuroblasts in mitosis
• Asymmetric Miranda localization requires an mRNA-dependent maintenance step

## Graphical Abstract

**Figure:**
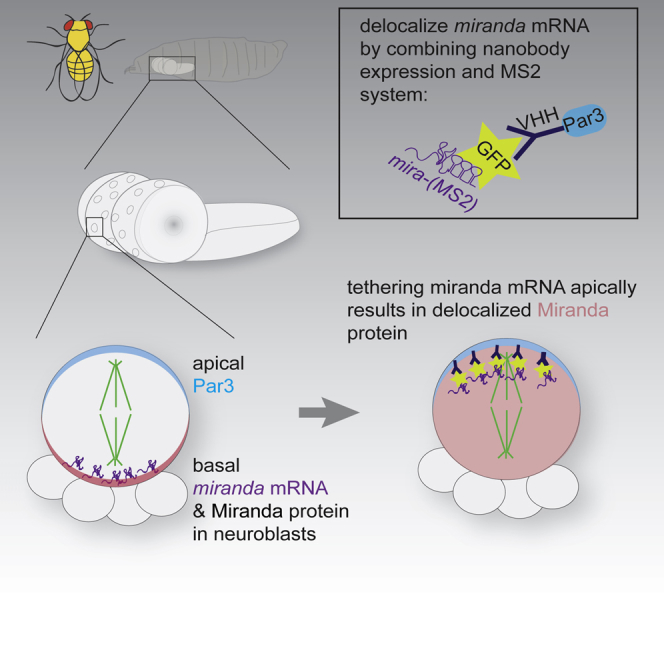

## Teaser

Ramat et al. combine the MS2 system and nanobody expression to alter the subcellular localization of mRNA. Shifting basally localized *miranda* mRNA in *Drosophila* neuroblasts to the apical pole resulted in Miranda protein localization defects. Miranda protein and cognate mRNA interaction positively feeds back on asymmetric Miranda localization.

## Introduction

A key problem for cells is to position their protein content correctly to ensure function in the right place. Positioning of proteins is complex, and it has become clear that one important element in this process is mRNA localization [1, 2, 3]. This raises the question how a given transcript governs the distribution of its protein product [4].

Genome-wide studies in *Drosophila* embryos revealed that transcript distribution frequently predetermined localization of the encoded proteins [5]. Moreover, the translation of mRNAs during transport to specific subcellular compartments is frequently repressed, which is lifted at the final destination [6, 7]. Therefore, mRNA localization and local control of translation are important factors influencing protein distribution.

However, the role of mRNAs is not limited to being the source of protein production. An emerging body of evidence suggests that coding mRNAs can have independent functions [8]. In zebrafish, Squint (Sqt), a Nodal-related signaling molecule belonging to the transforming growth factor β (TGF-β) superfamily, is involved in mesoderm induction and left-right axis specification. In addition, *sqt* mRNA can function in dorsal ventral axis specification [9]. During *Xenopus* development, *vegT* mRNA localizes to the vegetal cortex of the oocyte and seems to play a scaffolding role because oocytes depleted of VegT mRNA have a disorganized cytokeratin structure [10, 11]. Furthermore, during *Drosophila* oogenesis, the 3′ UTR of *oskar* (*osk*), a gene required for abdomen and germ cell formation [12], has a non-coding function that provides a scaffold to assemble ribonucleoprotein (RNP) complexes required for oocyte development [13]. Thus, mRNAs can provide essential non-coding functions that are linked to cell polarization and important for development.

Here, we address how mRNAs contribute to protein distribution in the context of asymmetrically dividing *Drosophila* neuroblasts (NBs). In these cells, fate determination depends on differential protein distribution at the cortex along the apico basal axis in preparation for division [14, 15]. At the apical pole, the Par complex, including aPKC, Par6, and Par3/Bazooka, assembles [16, 17, 18, 19]. This drives the basal localization of two adaptor proteins: Miranda (Mira) and Partner of Numb (Pon). This is important for basal localization and segregation of fate determinants, including Prospero and Numb to daughter cells, that are called ganglion mother cells (GMCs) [20, 21, 22, 23]. Whereas it has become clear that posttranslational modification of Mira is important to initiate its restricted localization basally [24, 25], how Mira localization is maintained through mitosis is unclear.

Intriguingly, many transcripts encoding for the molecular machinery behind NB asymmetry, including those of *mira*, show polarized distribution [5, 26, 27, 28, 29, 30, 31, 32]. The contribution of mRNA localization to NB polarity has only been marginally addressed. Mutation in *egalitarian* (*egl*; coding for a protein required for mRNA localization [33]) resulted in *insc* mRNA mislocalization in embryonic NBs, and *insc* mRNA doses were further found to be critical for correct execution of NBs division [27]. Loss of another RNA-binding protein Staufen (Stau) [34] was shown to affect *pros* mRNA localization [35], a condition that did not bring about any immediate defects, but when *pros* gene doses were simultaneously reduced led to problems in cell fate specification [36]. However, Egl and Stau are able to bind to several mRNAs [37, 38], limiting the use of mutation in these genes to address the role of the localization of transcripts from individual genes.

*mira* mRNA has been reported to localize apically in mitotic NBs, whereas Mira protein forms basal crescents in mitosis [26, 30]. Mutation in *mira* leads to cell fate transformation [39], which can trigger tumor-like growth of larval brains [40]. We therefore decided to address whether and how the localization of *mira* mRNA contributes to asymmetric Mira localization in mitosis.

We applied a variation of an approach used in cell culture cells to directly manipulate the localization of mRNA from a single gene [41]. Using genetically encoded tools, we were able to manipulate the subcellular localization of mRNA in NBs within the developing nervous system of *Drosophila*. We tagged endogenous *mira* mRNA with GFP using the MS2 system [42]. We then used nanobodies directed against GFP (hereafter GFP binding protein [GBP]), which, when fused to subcellular localization domains, can mislocalize GFP-tagged proteins [43]. We show that this can effectively redirect GFP-tagged mRNA in NBs using single-molecule fluorescent in situ hybridization (smFISH) [44] and use this to study *mira* mRNA localization in NBs.

## Results

### *mira* mRNA Localizes to the Apical Spindle Pole and in a Basal Crescent in Mitotic Neuroblasts

To address the role of *mira* mRNA localization, we developed methods to visualize it in living and fixed *Drosophila* larval brain NBs. Using gene editing, we generated an mRNA null mutant for *mira* by replacing part of its 5′ UTR and part of the first exon with an attP site. Animals homozygous for this allele (*mira*^*KO*^) die as embryos, as described for *mira* loss-of-function alleles [20]. Inserting the wild-type sequence into the attP site (*mira*^*WT-rescue*^) fully rescues (not shown) lethality of *mira*^*KO*^.

From this line, we derived various *mira* alleles by site-directed transgenesis [45] (see Figure S1). We further made a bacterial artificial chromosome (BAC) rescue construct for Mira, in which the protein was tagged with mCherry and the mRNA with MS2 stem loops in the 3′ UTR.

**Figure 1:**
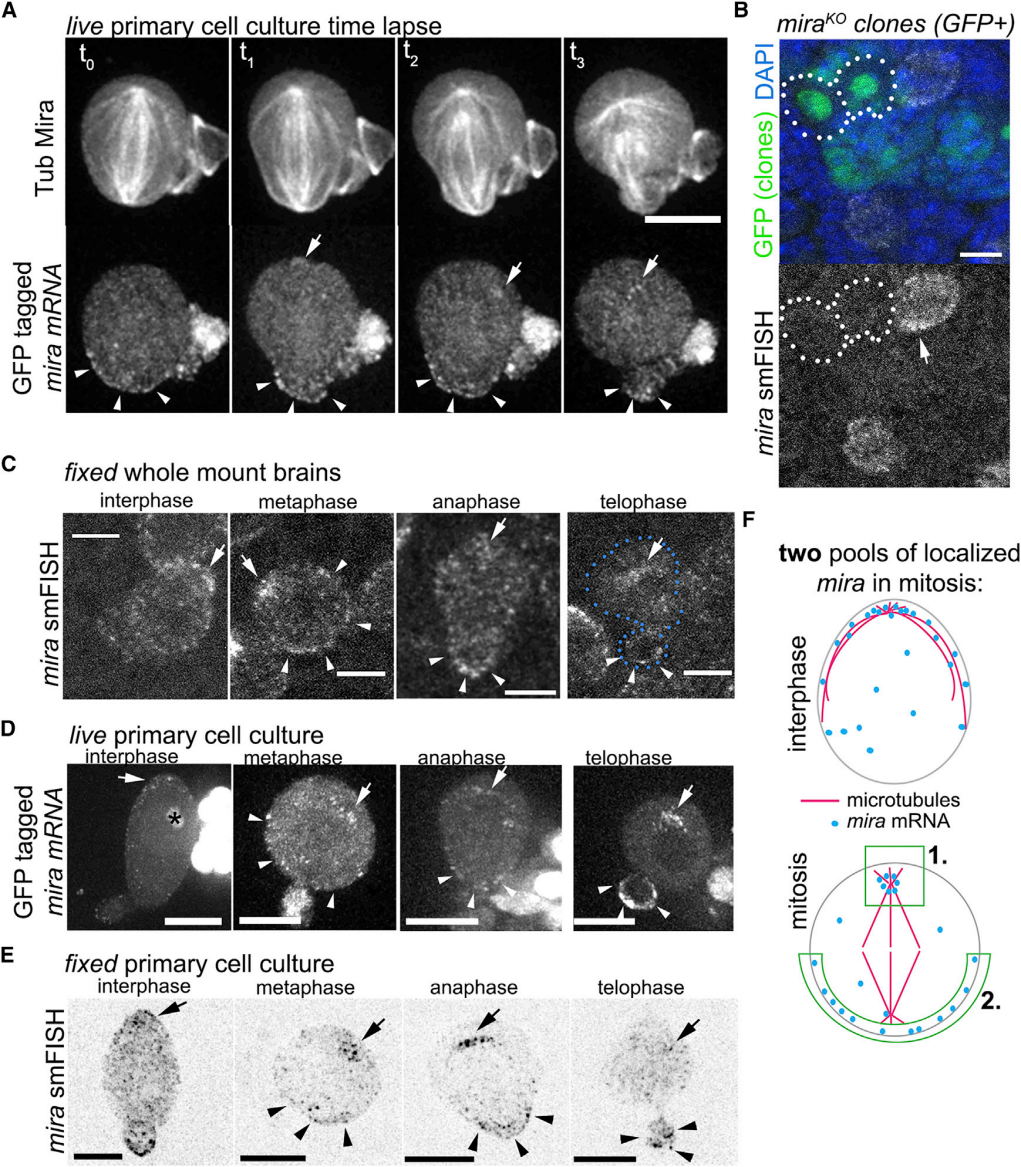
miranda mRNA Localizes in Two Different Pools in Mitotic Neuroblasts (A) Time-lapse imaging frames showing GFP-tagged *mira* mRNA (*wor-Gal4*, *UAS-MCP*::*GFP*; *BAC*{*mira*::*mCherry-*(*MS2*)}) in a NB in culture (related to Movie S3). Labels are as indicated. GFP spots are at the apical centrosome (arrow) and at the basal cortex (arrowheads), which are inherited by the GMC (t_3_, arrowheads). (B) *mira* smFISH on whole-mount brains harboring *mira*^*KO*^ homozygous mutant MARCM clones (GFP^+^). Dotted lines outline clones (arrow, *mira* smFISH signal in neighboring control cells). (C) *mira* smFISH on a *w*^*1118*^ brain. NBs at the indicated cell-cycle stages are shown. Arrowheads, basal *mira* mRNA crescents and *mira* segregating to GMCs; arrows, apically localized *mira* mRNA; dotted blue lines, NB outline at telophase. (D) GFP-tagged *mira* mRNA (*wor-Gal4, UAS-MCP::GFP; mira::mCherry-(MS2)*) in NBs in culture. The cell-cycle stage is indicated. Arrowheads, basal *mira* mRNA crescents; arrows, apical *mira* mRNA; asterisk, nucleolar MCP::GFP signal. (E) *mira* smFISH on NBs in culture. Arrowheads, basal *mira* mRNA crescents; arrows, apical *mira* mRNA. (F) Illustration of *mira* mRNA (blue dots) localization in NBs in interphase and mitosis. See also Figure S1. Scale bars indicate 10 μm.

In living whole-mount brains, we detected MCP::GFP apically enriched when *mira* mRNA carries MS2 stem loops (∼42% of NBs; n = 92), but not in controls (MCP::GFP carries a nuclear localization signal; Ctrl: Movie S1; no obvious GFP patterns; n = 23). In mitosis, GFP signal is readily detectable on the apical poles of spindles (Movie S2). In mitotic NBs in primary cell culture, where we can better select for NBs with lower MCP::GFP expression, GFP spots appear in a basal crescent that segregates to daughter cells (Figure 1A and related Movie S3; n = 15). Therefore, *mira* mRNA appears to localize in at least two different pools in living mitotic NBs.

To confirm that the GFP patterns correspond to *mira* mRNA, we used *mira* smFISH on fixed samples. These probes were specific because their signal dropped to background levels in clones for *mira*^*KO*^ (n = 5; Figure 1B). In control *w*^*1118*^ NBs in whole-mount brains, *mira* mRNA was apically enriched at the cortex in interphase. In mitosis, *mira* mRNA was found on the apical spindle pole and in a basal crescent in mitosis (Figure 1C). Similar localization patterns were observed in NBs in primary cell culture detecting *mira* mRNA using MCP::GFP (Figure 1D) and smFISH (Figure 1E).

Therefore, GFP-tagged *mira* mRNA and *mira* smFISH reveal similar distribution throughout the NB cell cycle in whole-mount brains and primary culture. We conclude that MS2-tagged *mira* faithfully reports *mira* mRNA localization and that at least two pools of localized *mira* mRNA can be distinguished in mitotic NBs (Figure 1F): *mira* mRNA localizes apically as previously described [26, 30]. Additionally, *mira* mRNA localizes in a basal crescent that segregates to daughter cells during NB division.

### *mira* mRNA Localization to the Apical Spindle Pole and to the Basal Cortex Is Differently Controlled

The identification of two pools of localized *mira* mRNA prompted us to address whether their localization mechanisms were the same. We therefore analyzed *egl* mutants, a gene involved in mRNA localization in *Drosophila* [33, 37] and *stau* mutants, because Stau is required for the basal localization of *pros* mRNA localization in NBs [35]. Given the localization around the apical spindle pole, we also tested whether microtubules were required for *mira* mRNA localization and whether preventing microtubule nucleation from the interphase centrosome by knocking down Centrobin (Cnb) [46] had any consequences for *mira* mRNA localization.

**Figure 2:**
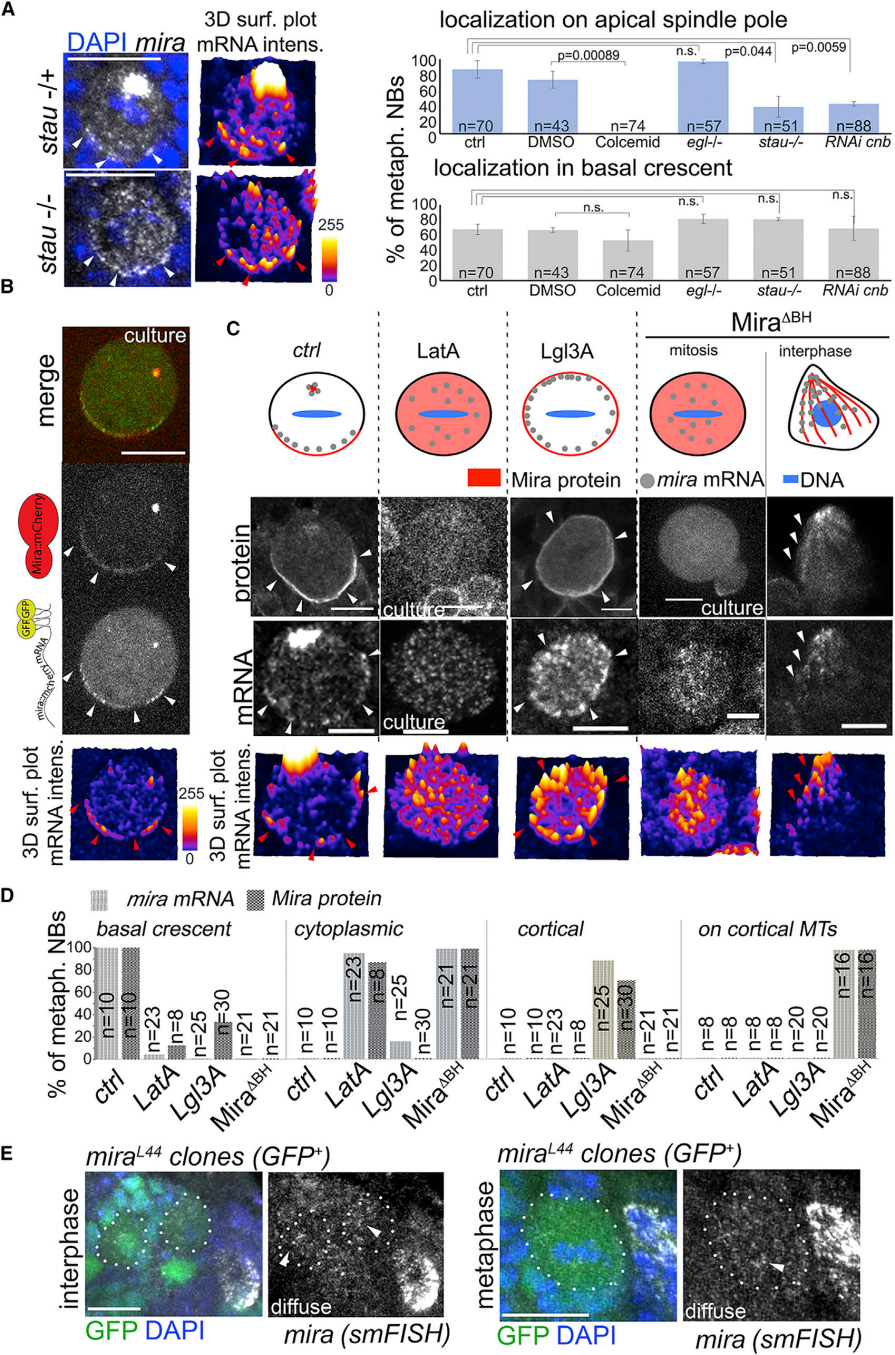
mira mRNA and Mira Protein Localization Patterns Are Spatially Correlated (A) Right: Quantification of the effect of the indicated conditions on *mira* mRNA localization to the apical spindle pole and basal crescents in mitotic NBs from whole-mount larval brains (colcemid [50 μM]). Error bars, SD. Left: *mira* smFISH on homo or heterozygous *stau*^*ry9*^ NBs. Arrowheads, basal *mira* crescents. (B) A *live* metaphase-arrested (50 μM colcemid) *BAC{mira::mCherry-(MS2)}* NBs expressing MCP::GFP by *wor-Gal4* (bright spot in cytoplasm likely to reflect a centrosome). Arrowheads, basal crescents of Mira protein and mRNA (related to Movie S4). (C) Top: schematic representation of Mira protein and mRNA localization under the indicated conditions. Middle: Mira protein localization detected with Mira::mCherry (or Mira antibody in ctrl and Lgl3A). Bottom: *mira* smFISH. Arrowheads, Mira protein and mRNA localization. LatA was used at 5 μM. Pictures showing protein and mRNA are from different cells, except for *mira*^*ΔBH*^*::mCherry* in interphase. (D) Frequency of the observed mRNA mislocalization patterns shown in (C). (E) smFISH using probes directed against *mira* mRNA on whole brains in which homozygous *mira*^*L44*^ mutant MARCM clones (GFP^+^) have been generated. Arrowheads, diffuse *mira* mRNA signal. Dotted lines outline mutant NBs. 3D surface plot represents mRNA signal intensities. See also Figures S1 and S2. Scale bars indicate 5 μm in (C) and 10 μm in (A), (B), and (E).

We find that *egl* is not essential for *mira* mRNA localization in NBs, as both pools remain detectable (Figures 2A and S2A). In contrast, removing Stau or knocking down Cnb appears to reduce *mira* mRNA localization to the apical spindle pole, but *mira* basal crescents remain unaffected (Figure 2A). Furthermore, *mira* mRNA localization to the apical spindle pole is highly sensitive to colcemid, whereas basal crescents are not (Figure 2A). Intriguingly, GFP-tagged *mira* redistributes to the basal NB pole upon microtubule depolymerization in living mitotic NBs in primary cell culture (Movie S4). Therefore, *mira* mRNA on the apical spindle pole is sensitive to loss of Stau, reduced Cnb levels, and microtubule depolymerization, whereas basal *mira* crescents are not. Thus, depending on where it localizes, *mira* mRNA might be differently controlled.

### *mira* mRNA and Protein Localization Is Spatially Correlated

Because NBs mutant for *stau* or expressing *cnb* RNAi disrupt *mira* localization on the apical spindle pole but do not have problems in terms of NB cortical polarity establishment [30, 46], we focused on *mira* mRNA localized in the basal crescent. We observed that Miranda protein and mRNA localization overlap at the basal pole (Figure 2B). Because, upon microtubule depolymerization, *mira* mRNA appears to relocate to the basal pole in mitotic NBs (Movie S4), *mira* mRNA appears to be attracted to localized Mira. We asked next whether *mira* mRNA localization always follows that of Mira protein.

We applied an approach used to alter the subcellular localization of GFP-tagged proteins involving GFP binding protein (GBP) fused to apically localized Bazooka (GBP::Baz) [43]. We generated homozygous Mira::GFP flies in which *mira* mRNA localizes in the two pools in NBs, as observed in *w*^*1118*^ in NBs (Figure S2B; n = 11 metaphase NBs from three optic lobes brains). In contrast, when we co-expressed GBP::Baz, defects in brain morphology are induced that are likely to reflect consequences of altered Mira segregation. Indeed, Mira::GFP is ectopically recruited to the apical pole of mitotic NBs, but *mira* mRNA is mostly cytoplasmic (Figure S2B; ∼80%; n = 20 metaphase NBs from four optic lobes). Therefore, *mira* mRNA localization is lost when Mira is force localized apically.

Force-localizing Mira by GBP::Baz might result, however, in abnormal NBs or alter the ability of Mira protein to interact with binding partners. We therefore sought to mislocalize Mira protein by other means. Correct Mira localization requires several factors, including an intact actin network [47] and the activity of aPKC [18]. Intriguingly, in mitotic NBs, Mira protein and mRNA are cytoplasmic upon actin network disruption or enriched on the entire NB cortex upon aPKC inhibition by Lgl3A overexpression [48] (Figures 2C and 2D). We also tested whether removing an important localization domain (BH motif) [24, 49] from Mira affects *mira* mRNA localization. We find that, when deleting the BH motif within Mira, Mira protein and mRNA become cytoplasmic in mitosis and both decorate specifically cortical microtubules in interphase (Figures 2C and 2D; see Movie S5). Thus, the localization of the mRNA follows the altered localization of the protein.

Finally, we tested whether *mira* mRNA localizes normally when it codes for an aberrant protein, unable to localize. To this end, we analyzed *mira* mRNA localization in *mira*^*L44*^ homozygous mutant NB clones. In these mutants, mRNA is produced, but due to a frameshift mutation, an altered protein results that is unable to localize [50]. In homozygous mutant *mira*^*L44*^ NB clones, the *mira* mRNA is diffusely localized (n = 23; Figure 2E). Therefore, *mira* mRNA localization appears to be determined by Mira protein.

### Expression of GFP-Binding Protein Fused to a Subcellular Localization Domain in Neuroblasts Efficiently Redirects GFP-Tagged mRNA

The finding that the mRNA follows the localization of the protein could indicate that the mRNA plays a role in localizing the protein. To test this, we sought to mislocalize the mRNA and measure the effects on Mira protein localization in NBs by combining the MS2 and GBP approaches.

**Figure 3:**
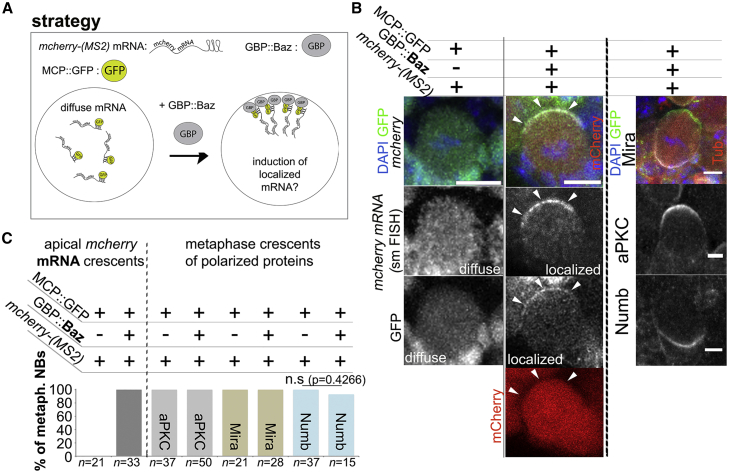
Efficient Induction of GFP-Tagged mRNA Localization by Subcellular Expression of GBP Fused to a Localization Domain (A) Strategy to mislocalize GFP-tagged *mcherry* mRNA (*mcherry-(MS2)*) to the apical pole in mitotic NB using GBP::Baz (see Figure S1 for details). (B) NBs in whole-mount brain preparations. Relevant genetic elements and labels are as indicated. Arrowhead, apically recruited *mcherry* mRNA and GFP (note the mCherry protein is not recruited apically). Right row: polarity markers are unaffected by tethering *mcherry* mRNA apically. (C) Left: quantification of the efficiency of *mcherry* mRNA tethering to the apical pole by GBP::Baz. Right: quantification of the effect of *mcherry* mRNA apical tethering on cortical polarity markers (unpaired t test). All upstream activating sequence (UAS) constructs were driven by *wor-Gal4*. See also Figure S1. Scale bars indicate 10 μm for panels involving *mcherry* smFISH and 5 μm in panels showing cortical polarity markers.

We first assayed whether a GFP-tagged but unrelated mRNA with a diffuse localization pattern can be induced to localize apically (Figure 3A). To this end, we generated animals that express *mcherry-*(*MS2*) mRNA from the *mira* locus (see Figure S1).

In NBs that express MCP::GFP, *mcherry-MS2* mRNA, but not GBP::Baz, *mcherry* mRNA, is diffusely localized in mitosis (Figure 3B). Strikingly, in the presence of GBP::Baz, *mcherry* mRNA is induced to co-localize with GFP. This appears to be the apical pole because it opposes basal Mira protein crescents (100%; n = 17; Figures 3B and 3C).

In this condition, mCherry protein remains detectable in the cytoplasm but never formed apical crescents (0/51 NBs; Figure 3B). Importantly, NB polarity as measured by aPKC, Mira, and Numb antibody staining is unaffected (Figures 3B and 3C).

Thus, combining the MS2 system to tag mRNA with GFP and subcellular GBP expression can be used to ectopically position mRNA without perturbing NB cortical polarity.

### Tethering *mira* mRNA at the Apical Cortex of Mitotic Neuroblasts Affects Basal Mira Protein Localization

**Figure 4:**
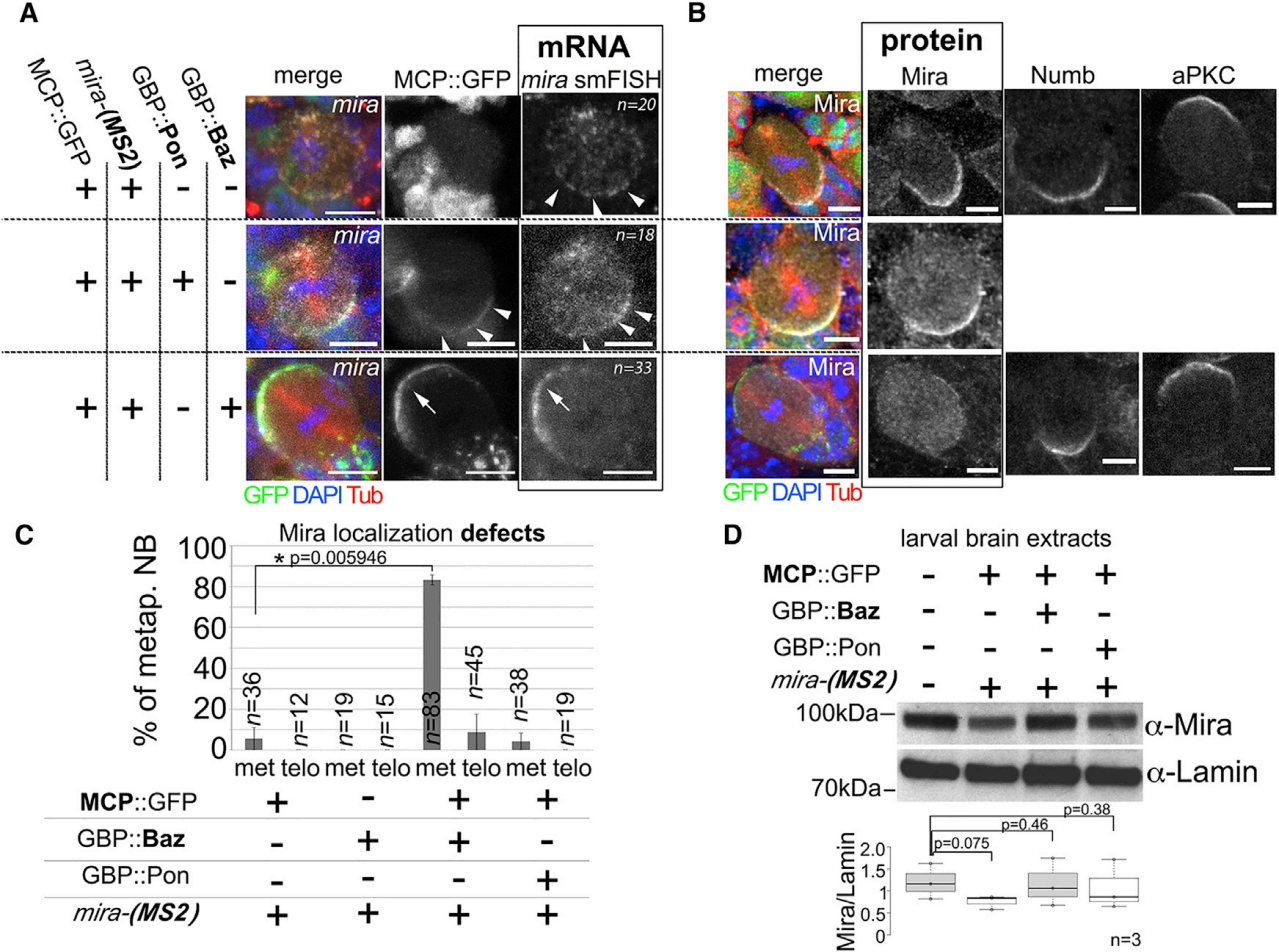
Tethering mira mRNA to the Apical Cortex in Mitosis Leads to Mira Protein Localization Defects (A and B) *mira* mRNA (A) and Mira protein and polarity marker localization (B) in NBs from *mira-*(*MS2*) homozygous brains in the indicated backgrounds. Arrowheads, basal localization of GFP and *mira* mRNA; arrows, apical localization of GFP and *mira* mRNA. (C) Quantification of Mira localization in metaphase (met) and telophase (telo) under the indicated conditions. See Figure S3A for quantification of the localization of the other markers shown in (B) (unpaired t test). (D) Western blot from larval brain extracts from animals of the indicated background. Quantification of Mira intensity relative to lamin is shown below (unpaired t test). Error bars, SD. All UAS constructs were driven by *wor-Gal4*. See also Figures S1 and S3. The scale bar indicates 5 μm.

We next applied this technique to NBs in which GFP-tagged *mira* transcripts are the only source of *mira* mRNA. In the absence of GBP, GFP-tagged *mira* mRNA localizes in the two pools and cortical polarity is unaffected (Figures 4A–4C and S3A). We first tested the effect of using GBP fused to the localization domain of PON to target MCP::GFP to the basal pole [43]. MCP::GFP now forms a basal crescent opposite to the mRNA pool on the apical spindle pole, but this has no apparent effect on *mira* mRNA localization, NB polarity, or Mira protein localization (Figures 4A–4C and S3A).

In contrast, in the presence of GBP::Baz, to mislocalize the mRNA to the apical NB pole, GFP forms apical crescents and so does *mira* mRNA, which largely depletes it from the basal pole of mitotic NBs. Whereas aPKC and Numb localization is similar to the control, Mira protein is unable to form a basal crescent and becomes mostly cytoplasmic (80% of metaphase NBs; n = 83; Figures 4A–4C and S3), which is rescued at telophase (Figure 4C). Absence of crescents in metaphase is unlikely to be caused by a gross reduction of Mira protein, because Mira levels as determined by western blot appear to be comparable between controls, in the presence of GBP::Pon or GBP::Baz (Figure 4D). Furthermore, cytoplasmic Mira levels appear to be elevated when the mRNA is tethered apically (Figure S3B). Thus, altering the localization of *mira* mRNA by GBP::Baz, albeit transiently, specifically affects Mira protein localization, which does not appear to be a consequence of compromised translation.

### Basal *mira* mRNA Maintains Mira Protein Localization in Mitosis

How could basal *mira* mRNA affect Mira protein localization? The mRNA might serve as a source for local translation. Alternatively, in *trans* interaction of *mira* mRNA and Mira protein might stabilize the localization of both at the basal cortex. To distinguish these possibilities, we sought to rescue Mira protein localization by adding *mira* mRNA lacking MS2 stem loops into the background that caused Mira protein localization defects and tested whether Mira protein localization was rescued. We chose *mira* mRNA from alleles, coding for protein unable to localize. If *mira* mRNA served as a local source of translation, Mira protein localization should not be rescued. If *mira* mRNA and protein crescents were restored, *mira* mRNA could be required independently of translation.

We tested two *mira* alleles that fulfill these criteria. We used *mira*^*L44*^ (containing a two-base insertion resulting in a frameshift and the production of an aberrant protein unable to localize [50]). We further engineered *mira*^*STOP*^ by substituting one base to generate an early stop codon. Homozygous *mira*^*stop*^ embryos, similar to those homozygous for *mira*^*KO*^, die at the end of embryogenesis. Importantly, *mira*^*STOP*^ expresses mRNA at levels comparable to controls. *mira*^*STOP*^ carries a hemagglutinin (HA) tag at the C terminus of the Mira coding frame, and HA antibody staining revealed a band of ∼70 kDa in brain extracts of heterozygous *mira*^*STOP*^ animals, likely to reflect a truncated protein initiated from a second ATG (positioned 648 bases downstream, which would have a predicted molecular weight of ∼66 kDa). The predicted truncated protein would lack the N-terminal region known to be required cortical association of Mira [50]. Consistently, HA staining on heterozygous *mira*^*STOP*^ larval brain NBs reveals only diffuse cytoplasmic signal, showing that this truncated protein is unable to localize in basal crescents, even in the presence of wild-type Mira protein (Figures S3C–S3F).

Only very few animals transheterozygous for *mira-(MS2)* and *mira*^*KO*^ (the necessary control background for the rescue experiment) progressed to larval stages in the presence of MCP::GFP and GBP::Baz, suggesting that insufficient Mira function remains in these animals. Therefore, we added an additional copy of MS2-tagged *mira* mRNA using the BAC rescue construct.

**Figure 5:**
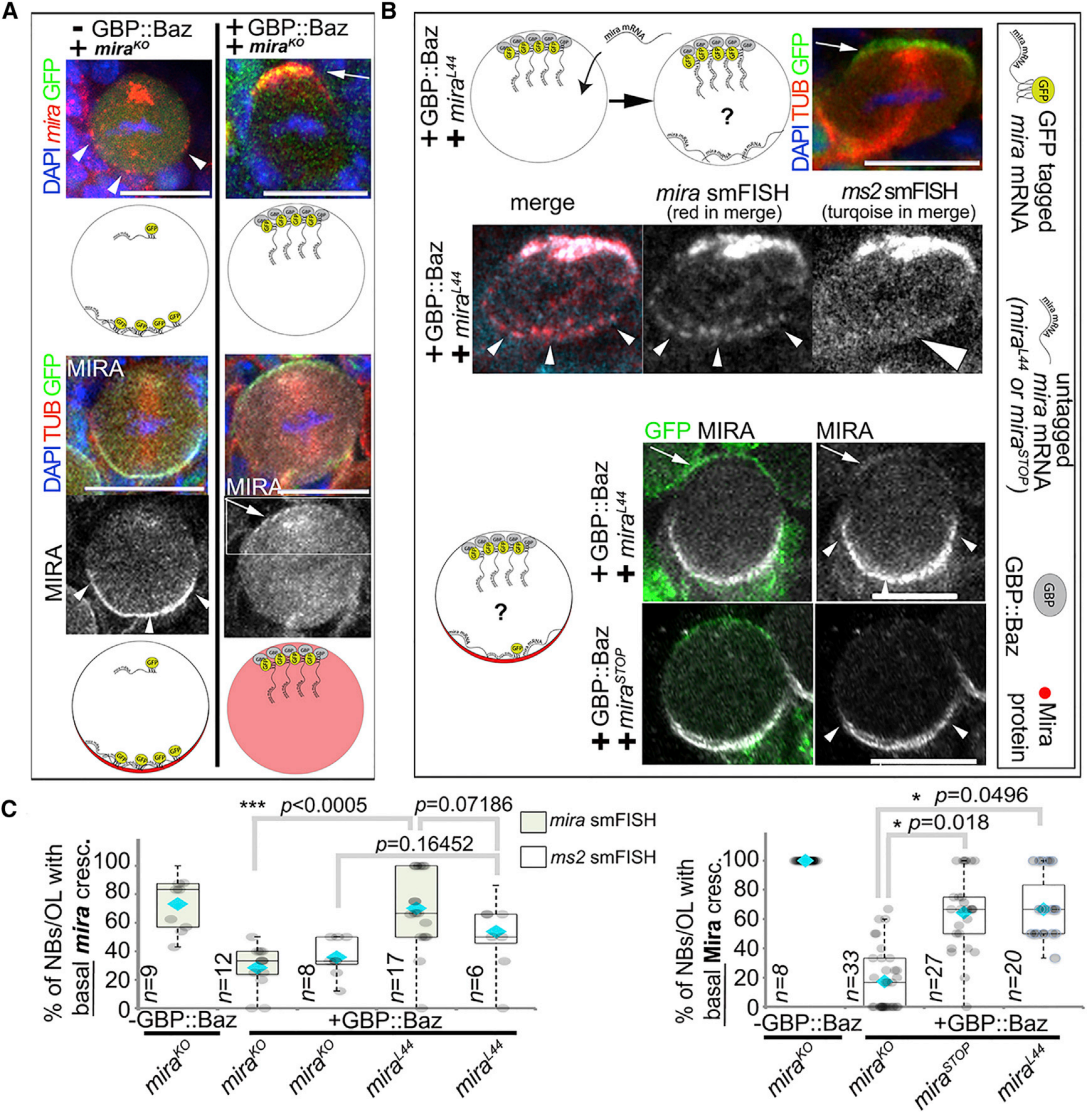
Mira Protein Localization Can Be Rescued by Untagged mira mRNA Encoded by a Different Allele (A) Transheterozygous *mira-(MS2)* and *mira*^*KO*^ NB expressing MCP::GFP in the absence or presence of GBP::Baz. First row: *mira* smFISH (red). Second row: cartoons of *mira* mRNA localization. Third row: effect on Mira protein localization. Fourth row: Mira channel alone (boxed area shown enlarged in Figure 6A). Arrow: apical *mira* mRNA and protein; arrowheads, basal *mira* mRNA and protein. (B) Protein localization rescue experiment. A cartoon of strategy is shown. A NB transheterozygous for *mira-(MS2)* and *mira*^*L44*^ expressing MCP::GFP and GBP::Baz, labeled for GFP, tubulin, DNA (shown in top row), and smFISH for *mira* and *ms2* (second row). Arrowheads, basal *mira* smFISH signal; large arrowhead, basal *ms2* smFISH signal. Third row: cartoon of experiment. NBs transheterozygous *mira-(MS2)* and *mira*^*L44*^ or *mira-(MS2)* and *mira*^*STOP*^ expressing MCP::GFP and GBP::Baz are shown. Arrowheads, basal Mira crescents; arrows, apical MCP::GFP and Mira protein. (C) Left: frequency of basal *mira* smFISH and *ms2* smFISH signal (*mira* smFISH quantification: −GBP, *mira*^*KO*^: 21 NBs/9 optic lobes [OLs]; +GBP, *mira*^*KO*^: 44 NBs/12 OLs; +GBP, *mira*^*L44*^: 21 NBs/17 OLs. *ms2* smFISH: +GBP, *mira*^*KO*^: 33 NBs/8 OLs; +GBP, *mira*^*L44*^: 23 NBs/6 OLs). Right: frequency of basal Mira protein crescents (−GBP, *mira*^*KO*^: 26 NBs/8 OLs; +GBP, *mira*^*KO*^: 115 NBs/33 OLs; +GBP, *mira*^*STOP*^: 108 NBs/27 OLs; +GBP, *mira*^*L44*^: 82 NBs/20 OLs). Error bars, SD. All NBs in this figure carry the *BAC*{*mira*::*mcherry-*(*MS2*)} construct on the second chromosome. Blue diamonds, average percentage; Mann-Whitney U test. See also Figures S1, S3, and S4. Scale bars indicate 10 μm.

Consequently, control animals for this experiment carry two alleles coding for *mira* mRNA that can be tagged with GFP and the mRNA null allele *mira*^*KO*^. In this background, in the presence of MCP::GFP, *mira* mRNA localizes normally in metaphase (n = 26 metaphase NBs from eight optic lobes; Figure 5A). Furthermore, adding GBP::Baz to mislocalize the mRNA apically efficiently redirects the mRNA to the apical pole (n = 44 metaphase NBs from 12 brain lobes; Figure 5A). As observed before (Figure 4), Mira becomes cytoplasmic in metaphase NBs (Figure 5A).

We then exchanged *mira*^*KO*^ with either *mira*^*L44*^ or *mira*^*STOP*^ to provide *mira* mRNA that cannot be apically tethered and tested whether basal mRNA localization would be restored. To distinguish between GFP-tagged and untagged mRNAs, we simultaneously used smFISH probes for all *mira* transcripts (*mira* smFISH, detecting MS2 tagged and untagged *mira* mRNA) and probes specific for the sequence harboring the MS2 stem loops (*ms2* smFISH; see also Figure S4). When we introduced *mira*^*L44*^, as expected, *mira* smFISH as well as *ms2* smFISH reveals strong overlapping signal at similar levels at the apical NB pole co-localizing with GFP (Figure 5B). Importantly, basal mRNA crescents become detectable and we detect significant *mira* smFISH signal, basally revealing individual dots. We also detected *ms2* signal basally, albeit in a more diffuse pattern (Figures 5B and 5C). Thus, basal mRNA crescents can be restored, and this pool is enriched for mRNA stemming from the *mira*^*L44*^.

As a consequence of introducing *mira*^*L44*^ or *mira*^*STOP*^, basal Mira protein localization was significantly rescued (Figures 5B and 5C). Therefore, it appears that providing mRNA that cannot be mislocalized by GBP contributes to restore basal Mira protein crescents, despite coding for protein unable to localize. These results prompt the possibility that Mira protein and mRNA can interact in *trans*.

### Mira Protein and mRNA Interact

**Figure 6:**
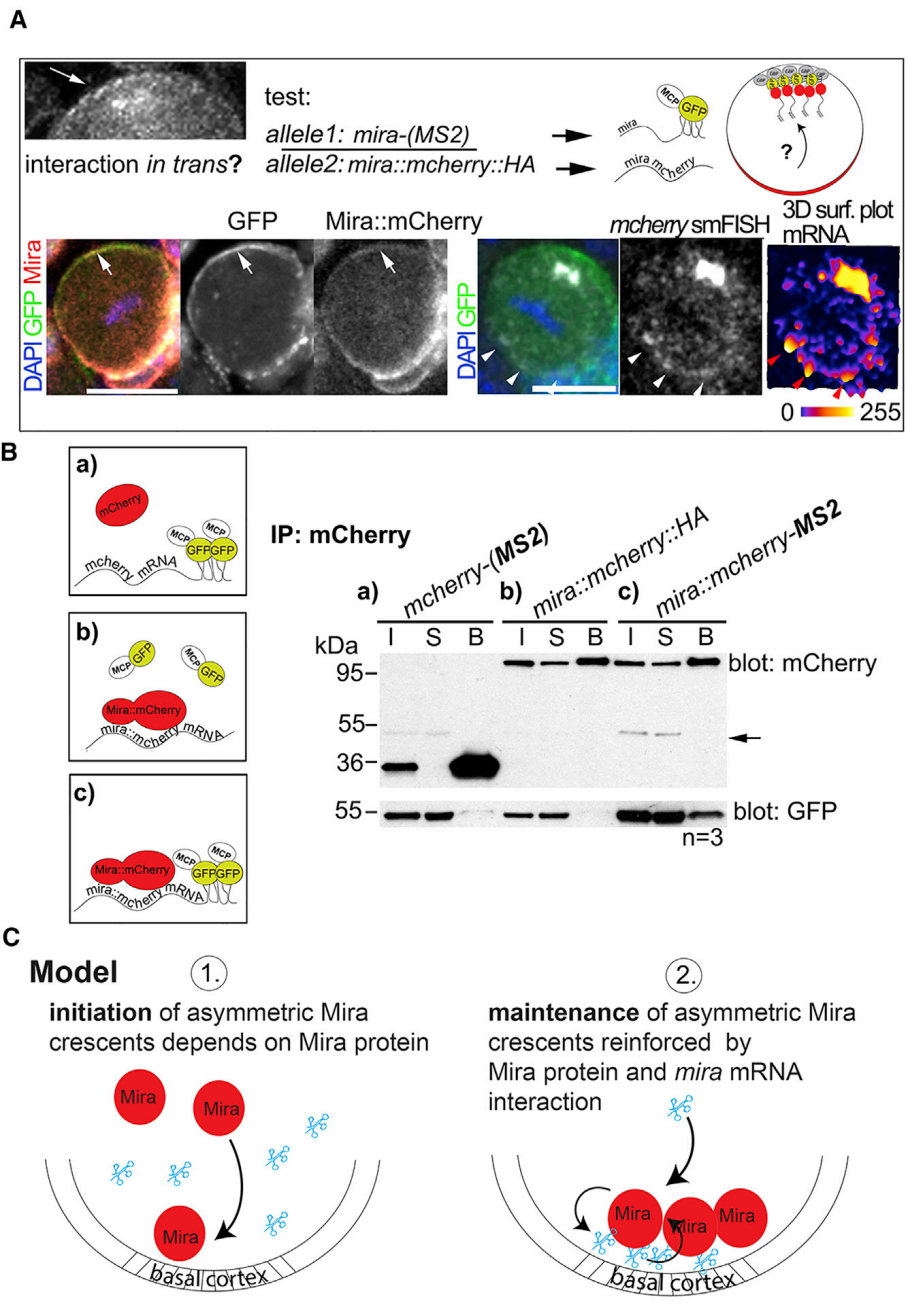
Mira Protein and mRNA Interact (A) Top left: inset of Figure 5A; arrow, apical Mira protein crescents induced by apically tethering the mRNA by which it is encoded. Top right: strategy used to test whether *mira* mRNA and protein interact in *trans*. A NB in a brain transheterozygous for *mira-(MS2)* and *mira::mcherry::HA* expressing MCP::GFP and GBP::Baz is shown. Arrows, Mira::mCherry can be detected apically co-localizing with GFP (42% of metaphase NBs; n = 21). *mcherry* smFISH on whole-mount brains of the same background. Arrowheads, *mcherry* signal is enriched basally. RNA signal intensities are shown in 3D surface plot. (B) Co-immunoprecipitation of MCP::GFP (mRNA) by mCherry from brain lysates. All samples express MCP::GFP. (a) *mcherry-(MS2)*, (b) *mira::mcherry*, and (c) *mira::mcherry-(MS2)* are shown. B, beads; I, 10% input; S, 10% supernatant. Arrow, residual GFP signal as blots were first probed for GFP. All UAS constructs were driven by *wor-Gal4*. (C) Model. See also Figure S1. The scale bar indicates 10 μm.

To test in *trans* interaction more directly, we made use of the observation that Mira forms weak apical crescents when *mira* mRNA was mislocalized apically (21/30 NBs; Figure 6A, inset). If Mira protein was able to interact in *trans* with cognate mRNA, tethering GFP-tagged *mira* mRNA coding for untagged Mira protein apically should recruit Mira::mCherry, produced by mRNA devoid of MS2 stem loops provided by another allele.

Indeed, we were able to detect faint Mira::mCherry crescents apically, whereas *mcherry* mRNA did not appear to be enriched apically in NBs transheterozygous for *mira::mcherry::HA* and *mira-(MS2)* (Figure 6A). Therefore, Mira::mCherry can be recruited to *mira* mRNA encoded by a different allele.

We further tested whether Mira protein and mRNA can be found in a complex. Indeed, Mira::mCherry can co-immunoprecipitate MCP::GFP, depending on MS2 stem loops in the mRNA (Figure 6B), supporting the notion that Mira mRNA and protein can be found in a complex. These results suggest that normally *mira* mRNA may contribute to maintain Mira protein basally through in *trans* interaction, which may be direct or require further factors.

## Discussion

In this study, we address how localized mRNA contributes to protein distribution during asymmetric division of *Drosophila* NBs. We demonstrate that combination of the MS2 system and subcellular nanobody (GBP) expression can be used to redirect mRNA within cells to study the function of mRNA localization (Figure 3). Using this approach, we reveal a mechanism that operates in NBs to maintain asymmetric distribution of Mira.

In NBs, *mira* mRNA localizes in two distinguishable pools. It segregates asymmetrically on the mitotic spindle (Figure 1), the function of which is unclear.

*mira* mRNA localization in a basal crescent (Figure 1) seems to be functionally important, however, because directing it away from the basal cortex results in Mira localization defects in mitosis (Figure 4). These defects do not appear to be caused by a gross reduction in protein levels (Figure 4). Consistently, Mira localization is restored at telophase, suggesting that then Mira levels are normal. Restored Mira asymmetry might be caused by the telophase rescue phenomenon [51, 52]. However, Baz that is apically localized in mitosis also redistributes basally at telophase (M.H. and J.J., unpublished data). Because mislocalization of *mira* mRNA is induced with a GBP::Baz fusion, the efficiency of *mira* mRNA removal from the basal cortex might be less efficient as mitosis progresses.

*mira* mRNA is, however, unable to localize in the absence of Mira protein (Figure 2E). Thus, rather than positioning the protein, the mRNA functions in maintaining Mira localization. Mira might be stabilized by in *trans* interaction with the cognate mRNA at the basal pole through positive feedback (Figure 6C). A similar mechanism operates also in the *Drosophila* oocyte, where Oskar protein regulates the localization of the cognate mRNA through positive feedback [53]. This view is supported by our finding that Mira can be ectopically recruited to mRNA provided by a different allele (Figure 6A) and that both can be found in a complex (Figure 6B). How Mira protein and mRNA interact remains unclear. The *mcherry* mRNA with diffuse localization (Figure 3) contained the full-length 5′ and 3′ UTRs of *mira* (see Figure S1B), which are therefore not sufficient to mediate this interaction. Furthermore, the BH motif within Mira seems dispensable for protein and mRNA interaction (Figure 2C). Moreover, the interaction might occur directly or involve further binding partners, but Stau seems not to be mediating this because mira mRNA crescents are detectable in *stau* mutant NBs (Figure 2A) that do not show Mira localization defects [30].

We never detected apical mCherry protein crescents when *mcherry* mRNA was tethered apically (Figure 3B), suggesting that mCherry protein rapidly diffuses away from its clustered mRNA. However, this is different for Mira, which can be detected in faint crescents at the apical pole when its mRNA is tethered there (Figure 6A). Phosphorylation by aPKC prevents the ability of Mira to be retained at the apical cortex [24]. Therefore, the faint crescents are likely to be a consequence of interaction with the apically tethered cognate mRNA. Apical crescents may then appear weaker as normal basal crescents, because aPKC activity might break the positive feedback apically. Alternatively, GBP-tethered *mira* mRNA may not be efficiently able to interact with Mira protein.

Our data cannot rule out a contribution of local translation to basal Mira crescents. The *mira*^*L44*^ and *mira*^*STOP*^ alleles used are translated. Moreover, we detect also mRNA coding for wild-type Mira protein basally in the rescue experiments (Figures 5B and 5C) and the interaction of Mira protein in the biochemical assay (Figure 6B) may reflect interaction due to the process of translation. Given that Mira localizes in crescents only for a few minutes in mitosis, it remains unclear whether local translation would be an efficient way to increase Mira protein levels in that time frame, however.

In any case, asymmetric mRNA localization either by serving as a local source of translation or by reinforcing protein localization through in *trans* interaction of protein and cognate mRNA may contribute to ensure different levels of protein concentration in particular subcellular locations as a means to strengthen cell polarization in general.

## STAR★Methods

### Key Resources Table

**Table:** 

REAGENT or RESOURCE	SOURCE	IDENTIFIER
**Antibodies**		

Rabbit polyclonal anti-Miranda	C. Gonzalez	N/A
Rabbit polyclonal anti-aPKC (C-20)	Santa Cruz Biotechnology	Cat# sc-216
Guinea pig anti-Numb	J. Skeath	N/A
Rat monoclonal anti-HA (clone 3F10)	Roche	Cat# 11867423001
Rabbit polyclonal anti-Egl	R. Lehmann	N/A
Mousse monoclonal anti-GFP	Roche	Cat# 11814460001
Rabbit polyclonal anti-mCherry	Abcam	Cat# ab167453
Mousse monoclonal anti-Lamin (clone ADL101)	DSHB	Cat# ADL101 s
Mousse monoclonal anti-Tubulin (clone 12G10)	DSHB	Cat# 12G10
Rabbit polyclonal anti-β Actin	Sigma	Cat# A2066
Donkey anti-Rabbit IgG Alexa-647	Life Technologies	Cat# A21244
Donkey anti-Guinea pig IgG Alexa-647	Life Technologies	Cat# A21450
Donkey anti-Rat IgG Alexa-647	Invitrogen	Cat# A21247
F(ab’)2-Goat anti-Rabbit IgG HRP	Life Technologies	Cat# A24537
Goat anti-Rat IgG HRP	Life Technologies	Cat# A10549
F(ab’)2-Goat anti-Mouse IgG HRP	Life Technologies	Cat# A24518

**Bacterial and Virus Strains**		

NEB 5-alpha Competent *E. coli*	NEB	Cat# C2987I

**Chemicals, Peptides, and Recombinant Proteins**		

Collagenase from *Clostridium histolyticum*	Sigma	Cat# C0130
Fibrinogen from human plasma	Sigma	Cat# F3879
Thrombin from bovine plasma	Sigma	Cat# T7513
Insulin from bovine pancreas	Sigma	Cat# I0516
Colcemid - CAS 477-30-5	Calbiochem	Cat# 234109
Latrunculin A	Sigma	Cat# L5163
RiboLock RNase Inhibitor	Thermo Scientific	Cat# EO0381
RFP-Trap_MA	Chromotek	Cat# rtma
cOmplete Protease Inhibitor Cocktail	Roche	Cat# 11697498001
Formaldehyde solution	Sigma	Cat# F8775

**Critical Commercial Assays**		

Gibson Assembly MasterMix	NEB	Cat# E2611
NucleoSpin RNA XS	Macherey-Nagel	Cat# 740902
qScript cDNA Synthesis Kit	VWR	Cat# 733-1173
PerfeCTa SYBR Green FastMix	VWR	Cat# 733-1381

**Experimental Models: Organisms/Strains**		

*D. melanogaster*: *w*^*1118*^	Bloomington Drosophila Stock Center	BDSC: 3605; Flybase: FBst0003605
*D. melanogaster*: RNAi of Cnb	Vienna Drosophila Resource Centre	VDRC: P{GD11735}v28651; Flybase: FBst0457594
*D. melanogaster*: MCP::GFP under UAS promoter	[54]	N/A
*D. melanogaster*: Gal4 under *worniu* promoter	[55]	N/A
*D. melanogaster*: mCherry::Jupiter under UAS promoter	C. Doe	N/A
*D. melanogaster*: FRT82B mira^L44^	[50]	Flybase: FBal0082443
*D. melanogaster*: Line to generate MARM clones *HS-Flp, UAS-GFPnls, tubulin-Gal4; FRT82B Gal80*	[56]	N/A
*D. melanogaster*: *baz::GFP*	[57]	N/A
*D. melanogaster*: *stau*^*RY9*^	D. St Johnston	Flybase: FBal0032815
*D. melanogaster*: *egl*^*WU50*^	[33]	N/A
*D. melanogaster*: *egl*^*PR29*^	[33]	N/A
*D. melanogaster*: Lgl3A::GFP under UAS promoter	[48]	N/A
*D. melanogaster*: GBP::Baz under UAS promoter	[43]	N/A
*D. melanogaster*: GBP::Pon under UAS promoter	[43]	N/A
*D. melanogaster*: BAC{mira::mcherry-MS2}	This paper	N/A
*D. melanogaster*: *mira*^*KO*^	This paper	N/A
*D. melanogaster*: *mira*^*KO*^	This paper	N/A
*D. melanogaster*: *mira*^*WT*^	This paper	N/A
*D. melanogaster*: *mira-(MS2)*	This paper	N/A
*D. melanogaster*: *mira::mcherry-(MS2)*	This paper	N/A
*D. melanogaster*: *mira::mcherry::HA*	This paper	N/A
*D. melanogaster*: *mira*^*STOP*^	This paper	N/A
*D. melanogaster*: *mira*^*ΔBH*^*::mcherry*	This paper	N/A
*D. melanogaster*: *mcherry-(MS2)*	This paper	N/A
*D. melanogaster*: *mira::GFP*	This paper	N/A

**Oligonucleotides**		

Forward primer for *mira* amplication in qPCR: CCATGTGGATCAGTTGAAGG	This paper	N/A
Reverse primer for *mira* amplication in qPCR: ATTCTCACTGGTCAGGGCTT	This paper	N/A
Forward primer for *tubulin* amplication in qPCR: TGTCGCGTGTGAAACACTTC	[58]	N/A
Reverse primer for *tubulin* amplication in qPCR: AGCAGGCGTTTCCAATCTG	[58]	N/A

**Recombinant DNA**		

pSL-MS2-12X	[42]	Addgene #27119
BAC CH322-11P04	BAC PAC Resources	CH322-11P4
RIV^white^	[59]	N/A
eTC GFP beta-actin full length	[60]	Addgene #27123
pTriEx-mCherry::LANS4	Kuhlman B.	Addgene # 60785

**Software and Algorithms**		

Fiji	[61]	https://fiji.sc/

### Contact for Reagent and Resource Sharing

Further information and requests for resources and reagents should be directed to and will be fulfilled by Jens Januschke (j.januschke@dundee.ac.uk).

### Experimental Model and Subject Details

#### Fly lines

Flies were raised on molasse-based food at 25°C. For whole mount brains and neuroblasts primary culture experiments, male and female early L3 larvae were used. Mitotic clones were generated by heat-shocking larvae 24h and 48h post hatching for 1 hr at 37°C. *w*^*1118*^ (Bloomington); UAS-MCP::GFP (M. Leptin [54]); worniu-Gal4 (C. Doe [55]); UAS-mCherry::Jupiter (C. Doe) FRT82B *mir*^*L44*^ (Matsuzaki [50])*;* hsFLP22, UAS-GFPnls, tubulin-GAl4; FRT82B gal80 [56]; UAS-cnb-RNAi (VDRC) ; Baz::GFP [57]; *stau*^*ry9*^ (D. StJohnston); *egl*^*WU50*^ and *egl*^*PR29*^ [33]; UAS-lgl3A::GFP (J. Knoblich [62]); UAS-GBP::Baz and UAS-GBP::Pon (M. Gonzalez-Gaitan [43]).

New mira alleles generated in this study (for details on their cloning and characteristics, see Method Details section): *BAC{mira::mcherry-MS2}; mira*^*WT*^; *mira-(MS2)*; *mira::mcherry-(MS2); mira::mcherry::HA*; *mira*^*STOP*^, *mira*^***Δ****BH*^*::*mCherry, *mcherry-(MS2)* and *mira::eGFP*.

#### Genotypes per figure

##### Figure 1

• w^1118^
• hsFlp22 tub-Gal4 UAS-nls::GFP ;; mira^KO^ FRT82B/FRT82B Gal80
• wor-Gal4 UAS-MCP::GFP; mira::mcherry-(MS2)
• wor-Gal4 UAS-MCP::GFP UAS-tub::mcherry ; BAC{mira::mcherry-(MS2)} Df (3R)ora^I9^, e

##### Figure 2

• w^1118^
• wor-Gal4
• egl^PR29^/egl^WU50^
• stau^ry9^
• wor-Gal4 / UAS-cnb RNAi
• wor-Gal4 UAS-MCP::GFP; BAC{mira::mcherry-(MS2)})} Df (3R)ora^I9^, e
• Baz::GFP ;; BAC{mira::mcherry-(MS2)}
• wor-Gal4/UAS-lgl3A::GFP
• mira^ΔBH^::mcherry/ mira^ΔBH^::mcherry
• hsFLP22 tub-Gal4 UAS-nls::GFP ;; mira^L44^ FRT82B/FRT82B Gal80

##### Figure 3

• wor-Gal4 UAS-MCP::GFP; mcherry-(MS2)
• wor-Gal4 UAS-MCP::GFP UAS-tub::mcherry/UAS-GBP::Baz; mcherry-(MS2)
• wor-Gal4 UAS-MCP::GFP/UAS-GBP::Baz; mcherry-(MS2)

##### Figure 4

• wor-Gal4 UAS-MCP::GFP UAS-tub::mcherry ; mira-(MS2)/mira-(MS2)
• wor-Gal4 UAS-MCP::GFP UAS-tub::mcherry/UAS-GBP::Baz ; mira-(MS2)/mira-(MS2)
• wor-Gal4 UAS-MCP::GFP UAS-tub::mcherry/UAS-GBP::Pon ; mira-(MS2)/mira-(MS2)
• w^1118^

##### Figure 5

• wor-Gal4 UAS-MCP::GFP UAS-tub::mcherry/ BAC{mira::mcherry-(MS2)} ; mira-(MS2)/mira^KO^
• wor-Gal4 UAS-MCP::GFP UAS-tub::mcherry/ BAC{mira::mcherry-(MS2)} ; UAS-GBP::Baz ; mira-(MS2)/mira^KO^
• wor-Gal4 UAS-MCP::GFP UAS-tub::mcherry/ BAC{mira::mcherry-(MS2)} ; UAS-GBP::Baz ; mira-(MS2)/mira^L44^
• wor-Gal4 UAS-MCP::GFP UAS-tub::mcherry/ BAC{mira::mcherry-(MS2)} ; UAS-GBP::Baz ; mira-(MS2)/mira^Stop^

##### Figure 6

• wor-Gal4 UAS-MCP::GFP/UAS-GBP::Baz; mira-(MS2)/mira::mcherry::HA
• wor-Gal4 UAS-MCP::GFP; mcherry-(MS2)
• wor-Gal4 UAS-MCP::GFP; mira::mcherry
• wor-Gal4 UAS-MCP::GFP; mira::mcherry-(MS2)

#### Neuroblasts primary culture

Brains were dissected in collagenase buffer [800mg NaCl, 200mg KCl, and 5mg NaH2PO4, 100mg NaHCO3 and 100mg D(+)Glucose in 100ml ddH2O] and incubated for 20 min in collagenase (0.2mg/ml, Sigma C0130). Brains were then transferred into a drop of fibrinogen (10mg/ml, Sigma f-3879) dissolved in Schneider’s medium (SLS-04-351Q) on a 25mm Glass bottom dish (WPI). Brains were manually dissociated with needles before the fibrinogen was clotted by addition of thrombin (100U/ml, Sigma T7513). Schneider’s medium complemented with FCS, Fly serum and Insulin (Sigma I0516) was then added and cells were kept at RT for 1 hr. When mentioned in the text, drugs (DMSO, LatA 5μM and Colcemid 50μM) were dissolved in supplemented Schneider’s medium.

### Method Details

#### Immunohistochemistry

Brains were dissected in PBS 1X, fixed in 4% Formaldehyde (FA, Sigma F8775) for 20min at RT and washed 3X 10min in PBS-Triton 1% before incubation with primary antibody (overnight, 4°C). Brains were washed 3X 10min in PBS-Triton 1% and incubated for 1 hr at RT with secondary antibody. Before mounting in Vectashield (VectorLabs, H-1000), brains were incubated for 30min in 50:50 PBS/Glycerol. Primary antibodies: Rabbit anti-Miranda (1:250, gift from C. Gonzalez), Rabbit anti-aPKC (1:500, Santa Cruz), Guinea pig anti-Numb (1:500, gift from J.Skeath) and Rat anti-HA (1/500, ROCHE 3F10). Secondary antibodies: donkey anti-Rabbit Alexa-647 (1/1000, life technologies), donkey anti-Guinea Pig Alexa-647 (1/1000) and donkey anti-Rat Alexa-647 (1/1000). Microscopy was performed using a Leica-SP8 CLSM (60x Water objective, NA1.2). Data was processed and analyzed using FIJI [61]. In all cases the sample size *n* provided reflects all samples collected for one experimental condition, unless specified otherwise in the figure legends. Experimental conditions were repeated at least twice to account for technical and biological variation. For Figure 5C, data from the different genotypes was pooled and Mira basal crescents per optic lobe were counted blind.

#### smFISH

Whole mount brain: Brains were dissected in PBS 1X, fixed in 4% FA (Sigma F8775) for 1 hr, washed in PBS 1X and then permeabilized overnight in 70% ethanol. Brains were then washed 5min in wash buffer (WB: formamide, 2X SSC and DEPC water) and hybridized overnight at 45°C under shaking with 125nM probe (Stellaris) in hybridization buffer (HB: formamide, 2X SSC, dextran glucose and DEPC water). After removal of the HB, brains were incubated 30min in pre-heated WB, then 30min WB with DAPI and mounted (Pro-Long Gold antifade reagent, Molecular probes #P36934).

NBs primary culture: NB cultures were prepared as described above and washed 3X in PBS 1X and fixed for 30min in 4% FA. Cells were permeabilized overnight with 70% ethanol, quickly washed with WB before hybridization with probes for 4 hr at 45°C. Cells were washed 30min with WB and 30min with WB + DAPI before mounting (Pro-Long Gold antifade reagent).

Microscopy was performed using a Leica-SP8 CLSM (60x Water objective, NA1.2). Data was processed and analyzed using FIJI. In all cases the sample size *n* provided reflects all samples collected for one experimental condition, unless specified otherwise in the figure legends. Experimental conditions were repeated at least twice to account for technical and biological variation.

#### Live imaging

NB cultures were prepared as described above and imaged using a 100x OIL objective NA1.45 on a spinning disk confocal microscope. Data was processed and analyzed using FIJI. In all cases the sample size *n* provided reflects all samples collected for one experimental condition. Experimental conditions were repeated at least twice to account for technical and biological variation.

#### Cloning and recombineering

For the generations of the different constructions, pSL-MS2-12X (Addgene, #27119) was the source for MS2 stem loops, CH322-11P04 was the source for the *mira* sequences and eTC GFP beta-actin (Addgene, #27123) was the source for the *eGFP* sequences.

*BAC{mira::mcherry-MS2}*, obtained by BAC recombineering based on CH322-11P04. InDroso functional genomics (Rennes, France) was used to generate *mira*^*KO*^ by gene editing (see Figure S1). Sequences for *mira*^*WT*^; *mira-(MS2)*; *mira::mcherry-(MS2); mira::mcherry::HA*; *mira*^*STOP*^, *mira*^***Δ****BH*^*::*mCherry, *mcherry-(MS2)* and *mira::GFP* were cloned using Gibson assembly into the RIV^white^ vector [59]. These constructions were then injected (University of Cambridge, *Drosophila* Microinjection Services) with phiC31 integrase system using *mira*^*KO*^ as landing site.

For *mira-(MS2), mira::mcherry-(MS2)* and *mcherry-(MS2)*, MS2 loops sequences were added in-between the coding sequence and *mira* 3′UTR. For *mira*^***Δ****BH*^*::*mCherry, amino acids 72-110 of Mira are deleted. For *mira*^*STOP*^, Gibson assembly was used to induce a substitution and changing the 8^th^ N-terminal amino acid into a stop codon (TTG to TAG) and to add an HA tag into Mira C-terminal region.

#### Co-Immunoprecipitation

Brains were lysated in extraction buffer [25mM HEPES pH6.8, 50mM KCl, 1mM MgCl2, 1mM DTT, 125mM sucrose, protease inhibitor and RiboLock RNase Inhibitor (Thermo Scientific)]. After 10min centrifugation at 4°C, lysate was applied to RFP-Trap beads (ChromoTek) and incubate for 1h at 4°C on a rotative wheel. Beads were washed five times in RIPA buffer [50mM Tris-HCl pH7.5, 1% NP-40, 1% sodium desoxycholate, 0.1% SDS, 1mM EDTA and 1mM NaCl]. Samples were then processed for western blotting. Experimental conditions were repeated twice to account for technical and biological variation.

#### Western blotting

Samples were homogenized in RIPA extraction buffer [10 mM Tris/Cl (pH 7.5), 150 mM NaCl, 5 mM EDTA, 0.1% SDS, 1% Triton X-100, 1% Deoxycholate and protease inhibitor cocktail (Roche)]. Blots were probed with rabbit anti-Miranda (1/500), rat anti-HA (1/1000, ROCHE 3F10), rabbit anti-Egl (1/2500, gift from R. Lehmann), mousse anti-GFP (1/1000, ROCHE 11814460001), rabbit anti-mCherry (1/1000, Abcam ab167453), mouse anti-Lamin (1/500, DSHB ADL101), mouse anti-Tubulin (1/2000, DHSB 12G10) and rabbit anti-β-actin (1/3000, SIGMA) antibodies. HRP-conjugated secondary antibodies (anti-rabbit, -rat and -mouse from Life Technologies) were revealed by chemiluminescent detection (Pierce).

#### RNA extraction and qPCR

Quantitative PCR (Q-PCR) was performed on a Bio-Rad CFX Connect with PerfeCTa SYBR Green SuperMix (Quanta) on cDNA synthesized (using qScript cDNA Mix; Quanta) from 1 μg total RNA (Nucleospin RNA XS; Macherey-Nagel) extracted from stage 9-12 embryos. For each experiment, samples were made in triplicate and experiments were repeated at least twice to account for technical and biological variation.

### Quantification and Statistical Analysis

All experiments were at least done in three biological repeats that served as the basis for the statistical analysis. Unpaired t test and Mann-Whitney U test were performed in Microsoft Excel for Mac 2011. The data in Figure 5C compares the percentage of observed phenotypes in mitotic NBs per optic lobe. Since the number of mitotic NBs in optic lobes varies and cannot be controlled we assumed that the data was not normally distributed. While we scored a high number of NBs, the basis for the statistical testing were three biological repeats. Therefore we used the Mann-Whitney U test to reject the hypothesis that the distributions are identical.

## Author Contributions

A.R. and J.J. designed the experiments. A.R., M.H., and J.J. performed experiments. A.R., M.H., and J.J. interpreted the results. J.J. wrote the paper that was agreed upon by all authors.

## Supplemental Information

Document S1. Figures S1–S4

Movie S1. In the Absence of MS2-Tagged mRNA, the MCP::GFP Reporter Shows No Patterned Localization in the Cytoplasm, Related to Figure 1 A NB in a whole mount brain explant expressing MCP::GFP (green) by wor-Gal4. Tubulin labeled in red. Time resolution: 1 min.

Movie S2. MCP::GFP Detects MS2-Tagged *mira* mRNA on the Apical Spindle Pole, Related to Figure 1 A NB in a heterozygous BAC{mira::mCHerry-(MS2)} whole mount brain explant expressing MCP::GFP (green) by worniu-Gal4. Tubulin labeled in red Time resolution: 1 min.

Movie S3. *mira* mRNA Localizes in a Basal Crescent and Segregates to Daughter Cells during Division, Related to Figure 1 A NB in primary cell culture from whole mount brains heterozygous for heterozygous BAC{mira::mCHerry-(MS2)} and expressing MCP::GFP (red) by wor-Gal4. Tubulin labeled in blue. Time as indicated.

Movie S4. Upon Microtubule Depolymerization, *mira* mRNA Redistributes Basally in a Mitotic Neuroblast, Related to Figure 2 A NB in primary cell culture from whole mount brains heterozygous for heterozygous BAC{mira::mCHerry-(MS2)} expressing MCP::GFP (red) by *wor-Gal4*. Tubulin labeled in blue. The effect of colcemid is visible by the disappearance of spindle microtubules. Time resolution: 1 min.

Movie S5. Mira^ΔBH^::mCherry Localizes to Cortical Microtubules in Interphase, Related to Figure 2 A NB in primary cell culture expressing Mira^ΔBH^::mCherry and treated with 50μM colcemid at the beginning of the movie. Mira^ΔBH^::mCherry appears to localise to cortical microtubules since localized signal disappears upon colcemid treatment (15/19NBs). Time and scale as indicated.

Document S2. Article plus Supplemental Information
