# Supplementary material for: Maintenance of Miranda Localization in Drosophila Neuroblasts Involves Interaction with the Cognate mRNA
Source: Curr Biol. 2017 Jul 24;27(14):2101–2111.e5. doi: 10.1016/j.cub.2017.06.016 (PMC5526833; doi:10.1016/j.cub.2017.06.016)
Supplement: Document S1. Figures S1–S4 [file mmc1.pdf]

Current Biology, Volume *27*

Supplemental Information

Maintenance of Miranda Localization in *Drosophila* Neuroblasts Involves Interaction with the Cognate mRNA

Anne Ramat, Matthew Hannaford, and Jens Januschke

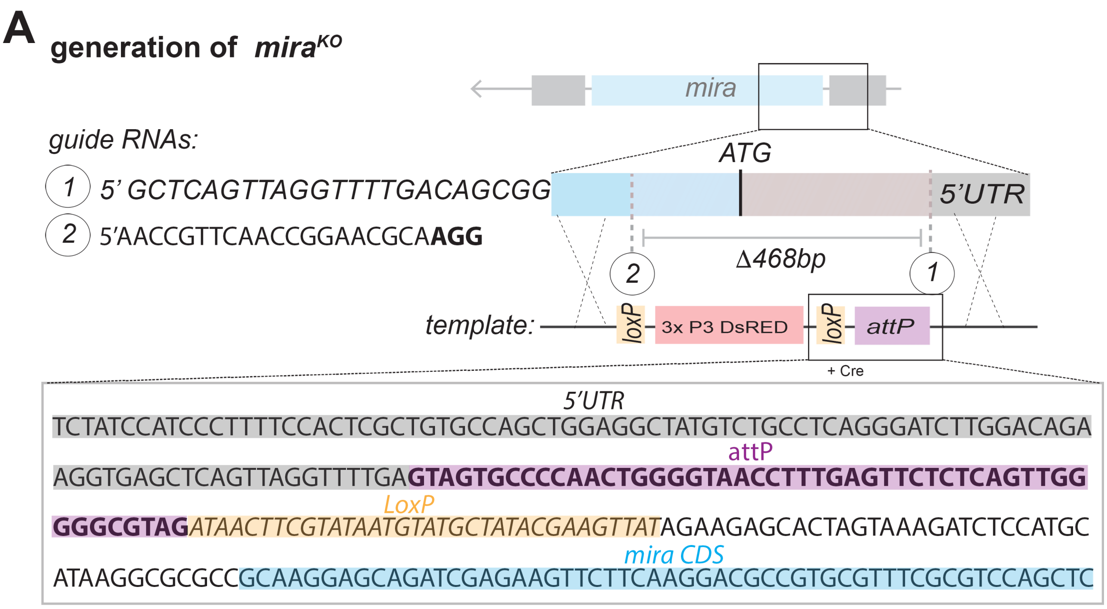

B alleles generated

allele name	mRNA features: FL 5' mira UTR	FL 3' mira UTR	MS2 stem loops	mRNA made?	protein features: mCherry tag	HA tag	protein made?	Mira localizes?	rescues embryonic lethality?	comment
BAC{mira::mcherry-(MS2)}	✓	✓	✓	✓	✓	–	✓	✓	✓	6 stem loops; **best for live** mRNA imaging, inserted in attP ZH-86Fb
mira::mcherry-(MS2)	✓	✓	✓	✓	✓	–	✓	✓	✓	6 stem loops; **best for live** mRNA imaging
mira^{KO}	–	–	–	–	–	–	–	–	–	mRNA and protein null allele, attP site within *mira* locus
mira$^{wt\text{-}rescue}$	✓	✓	–	✓	–	–	✓	✓	✓	reinserting an **untagged full length** genomic region fully rescues
mira::mcherry::HA	✓	✓	–	✓	✓	✓	✓	✓	✓	wild type Mira tagged with **mCherry** and **HA**
mira-(MS2)	✓	✓	✓	✓	–	–	✓	✓	✓	12 stem loops; **homozygous viable** in the presence of MCP-GFP in NBs
mcherry-(MS2)	✓	✓	✓	✓	✓	–	–	n.a.	–	5' and 3' UTR of *mira* fused to sequence coding for mCherry inserted into attP site at *mira* locus.
mira^{STOP}	✓	✓	–	✓	–	✓	✓	–	–	**truncated protein** made lacking cortical localization domain
mira^{L44}	✓	✓	–	✓	–	–	✓	–	–	**aberrant protein** made, that does not localize (Matsuzaki et al, 1998)
mira::GFP	✓	✓		✓	–	–	✓	✓	✓	wild type Mira tagged with **GFP**

Figure S1: Generation of *mira*^{KO} and the alleles derived from it. Related to Figures 1-6. (A) Illustration of the generation of *mira*^{KO} by CRISPR/Cas9 with the sequence of *mira*^{KO} obtained after the deletion. **(B)** Summary of different *mira* alleles used in this study.

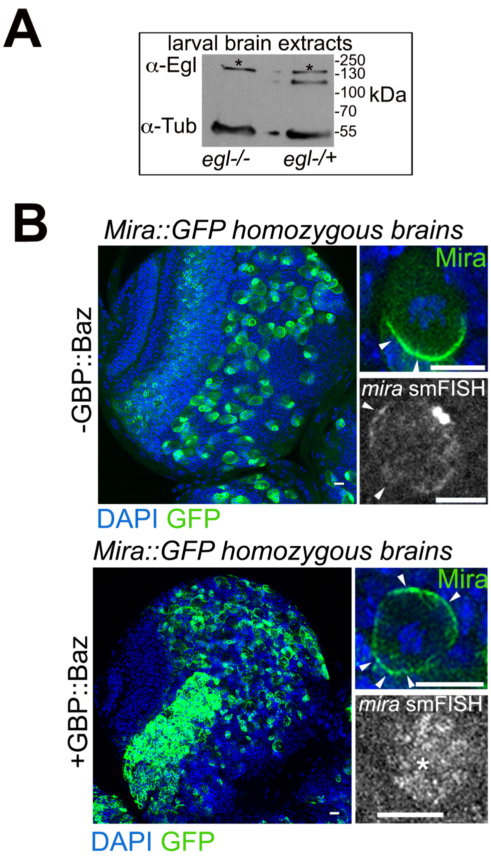

Figure S2: When Mira::GFP is tethered apically *mira* mRNA localizes to the cytoplasm. Related to Figure 2. (A) Inset: Egl (MW of ~ 112kDa) is detectable in egl^{WU50} or egl^{PR29} heterozygous (*egl*-/+), but not in egl^{WU50}/egl^{PR29} transheterozygous mutants (*egl*-/-). Asterisks: unspecific higher molecular weight band. **(B)** Optic lobes from whole mount brains of homozygous *mira::GFP* animals expressing *worniu*-Gal4 alone (top) or together with GBP::Baz (bottom). Insets show high power images of representative NBs in which *mira* mRNA was detected by smFISH (grey). Arrowheads: *mira* mRNA and protein (Mira::GFP) crescents in the control (-GBP::Baz). In the presence of GBP::Baz, arrowheads point at cortical Mira::GFP and note its present at the apical pole. Asterisks highlights diffuse cytoplasmic *mira* mRNA when Mira::GFP is mislocalized. Labels as indicated. Scale bars 10μm.

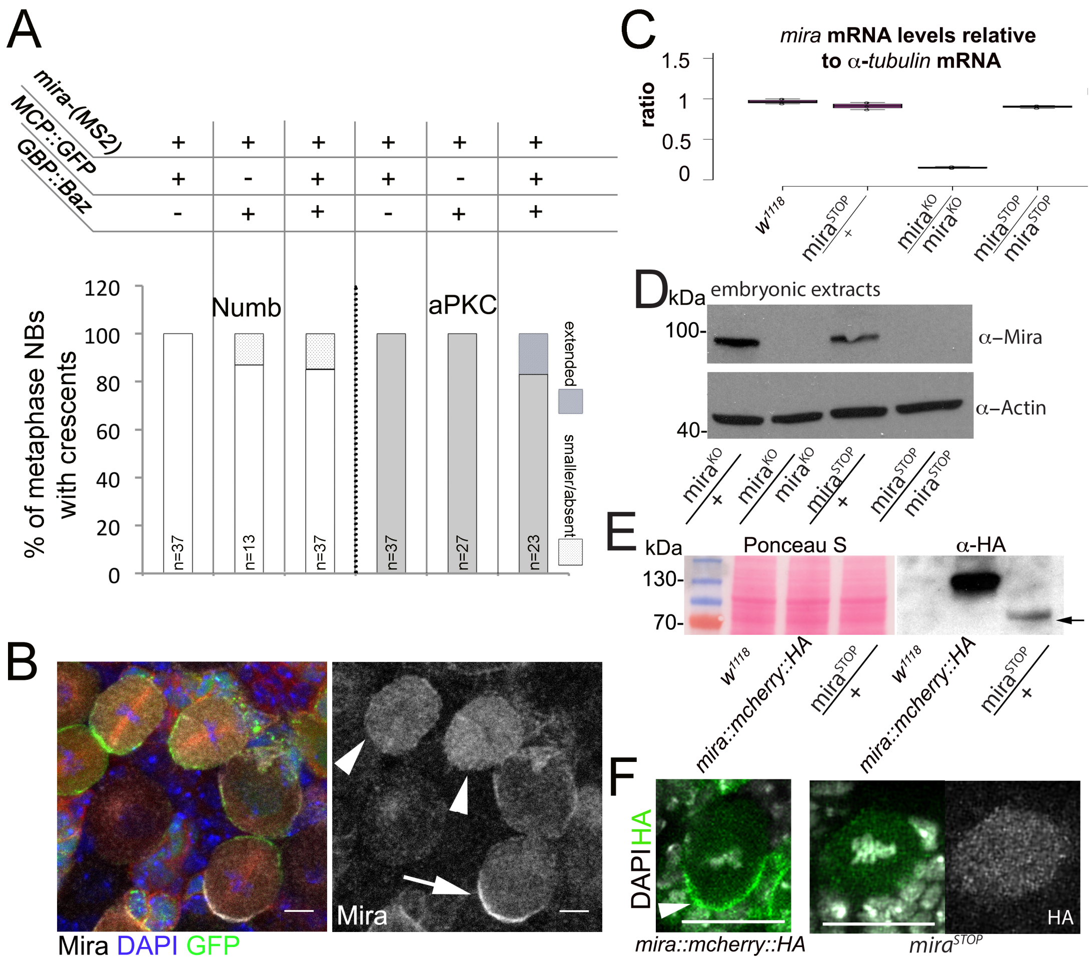

Figure S3: aPKC and Numb localization is not affected by directing endogenous *mira* mRNA to the apical pole. Related to Figure 4 and 5. (A) Quantification of Numb and aPKC crescents in mitotic NBs when *mira* mRNA is tether to the apical cortex. Genetic elements indicated on the top. **(B)** Low power magnification showing Mira protein (grey) localization defects when *mira* mRNA is tether to the apical cortex of mitotic NBs. Note that in NBs failing to localize Mira in a basal crescent (arrowheads), cytoplasmic Mira level is higher compared to a NB with a crescent (arrow). Scale bar 5µm. **(C)** Quantitative PCR showing *mira* mRNA levels relative to a-tubulin mRNA in extracts from embryos. Genotypes as indicated. Note that (1) $mira^{KO}$ mutant embryos have very low *mira* mRNA levels and (2) $mira^{STOP}$ have levels similar to controls. Error bars: standard deviation. **(D)** Western blot from embryo extracts to reveal Mira protein presence. Actin served as loading control. Genotypes as indicated. Note that Mira protein is not detected in homozygous $mira^{KO}$ and $mira^{STOP}$ mutant embryos. **(E)** Western blot using HA antibody. Ponceau S as loading control. Genotypes as indicated. HA antibody does not detect any protein in extracts from brains of w^{1118}, but detects a band corresponding to control

Mira::mCherry::HA (from heterozygous *mira::mcherry::HA*) and a ~70kD band from extracts of heterozygous *mira*^{STOP} brains. **(F)** Immunostaining for HA (green) of mitotic NBs heterozygous for *mira::mCherry::HA* or *mira*^{STOP} in whole mount brain preparations. HA detects a crescent (arrowhead) in *mira::mCherry::HA* but only diffuse staining in *mira*^{STOP} brains. Scale bar 10µm.

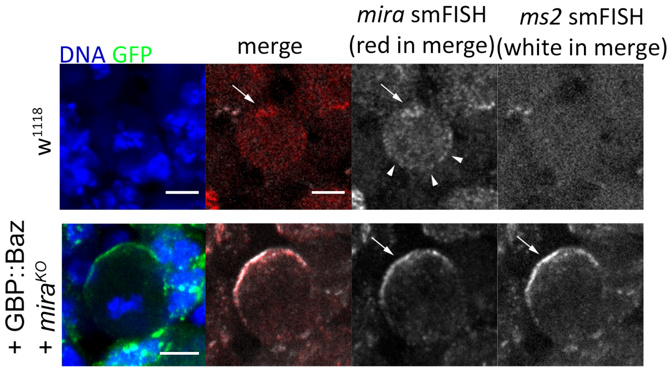

Figure S4: Specificity of *ms2* smFISH probes. Related to Figure 5. (A) NBs from whole mount brain preparation double labeled with *mira* smFISH and *ms2* smFISH. In w^{1118} NBs, while *mira* smFISH is detected in two pools, *ms2* smFISH revealed no signal. In NBs expressing GBP::Baz and heterozygous for *mira-(MS2)* and *mira*^{KO} both probes detect signal at the apical pole. Scale bar, 10µm.
