# Supplementary figures and images for: Maintenance of Miranda Localization in Drosophila Neuroblasts Involves Interaction with the Cognate mRNA
Source: Curr Biol. 2017 Jul 24;27(14):2101–2111.e5. doi: 10.1016/j.cub.2017.06.016 (PMC5526833; doi:10.1016/j.cub.2017.06.016)

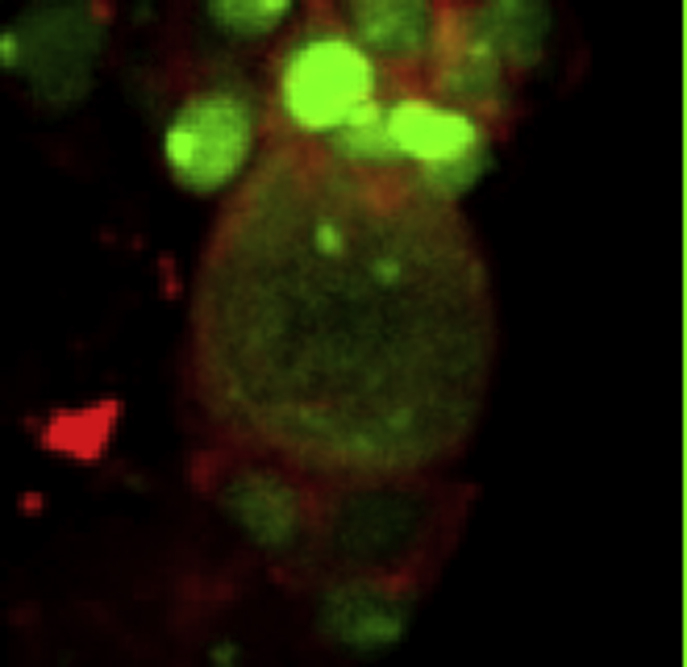

Supplement: Movie S1. In the Absence of MS2-Tagged mRNA, the MCP::GFP Reporter Shows No Patterned Localization in the Cytoplasm, Related to Figure 1 — A NB in a whole mount brain explant expressing MCP::GFP (green) by wor-Gal4. Tubulin labeled in red. Time resolution: 1 min. [file mmc2.jpg]

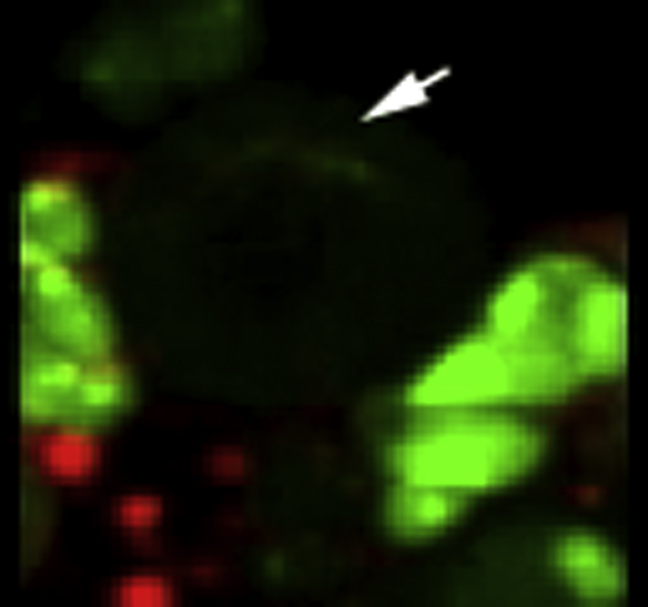

Supplement: Movie S2. MCP::GFP Detects MS2-Tagged mira mRNA on the Apical Spindle Pole, Related to Figure 1 — A NB in a heterozygous BAC{mira::mCHerry-(MS2)} whole mount brain explant expressing MCP::GFP (green) by worniu-Gal4. Tubulin labeled in red Time resolution: 1 min. [file mmc3.jpg]

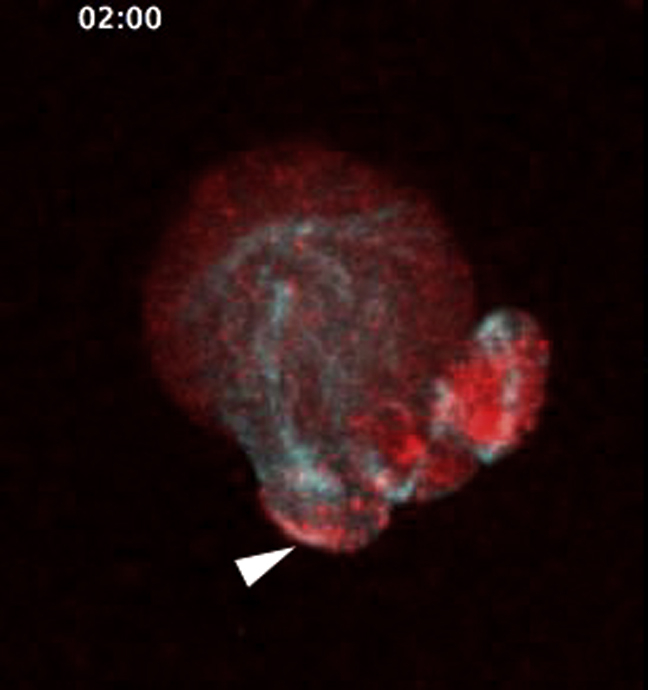

Supplement: Movie S3. mira mRNA Localizes in a Basal Crescent and Segregates to Daughter Cells during Division, Related to Figure 1 — A NB in primary cell culture from whole mount brains heterozygous for heterozygous BAC{mira::mCHerry-(MS2)} and expressing MCP::GFP (red) by wor-Gal4. Tubulin labeled in blue. Time as indicated. [file mmc4.jpg]

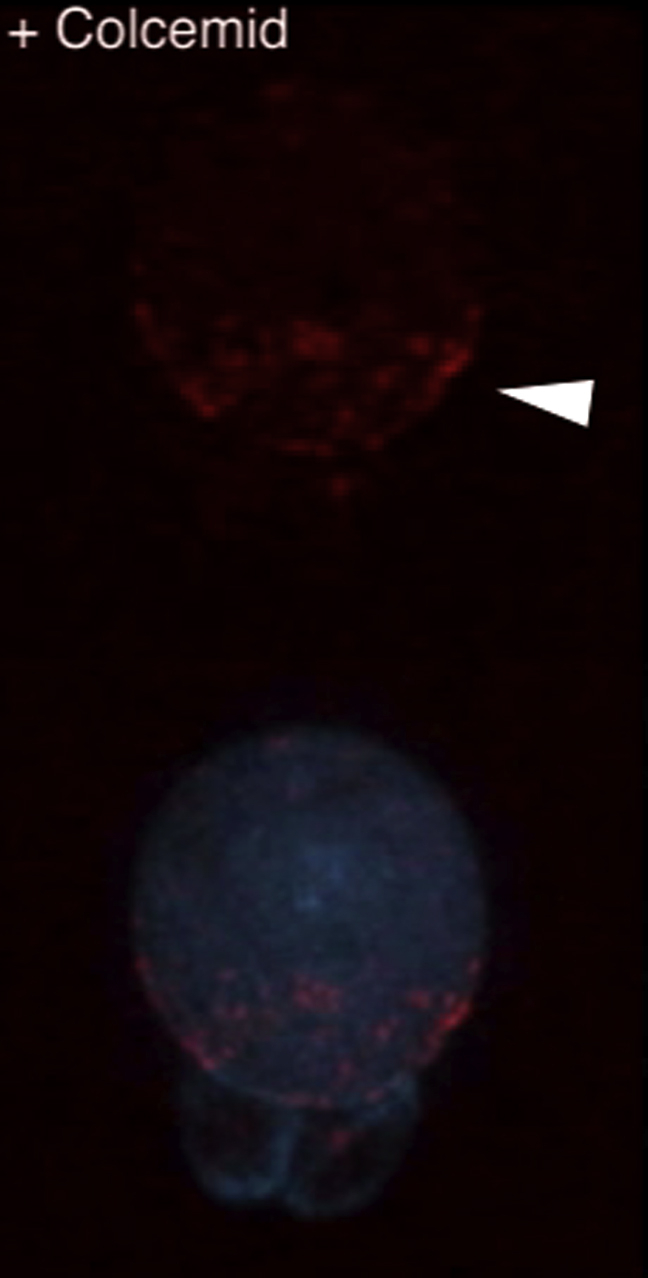

Supplement: Movie S4. Upon Microtubule Depolymerization, mira mRNA Redistributes Basally in a Mitotic Neuroblast, Related to Figure 2 — A NB in primary cell culture from whole mount brains heterozygous for heterozygous BAC{mira::mCHerry-(MS2)} expressing MCP::GFP (red) by wor-Gal4. Tubulin labeled in blue. The effect of colcemid is visible by the disappearance of spindle microtubules. Time resolution: 1 min. [file mmc5.jpg]

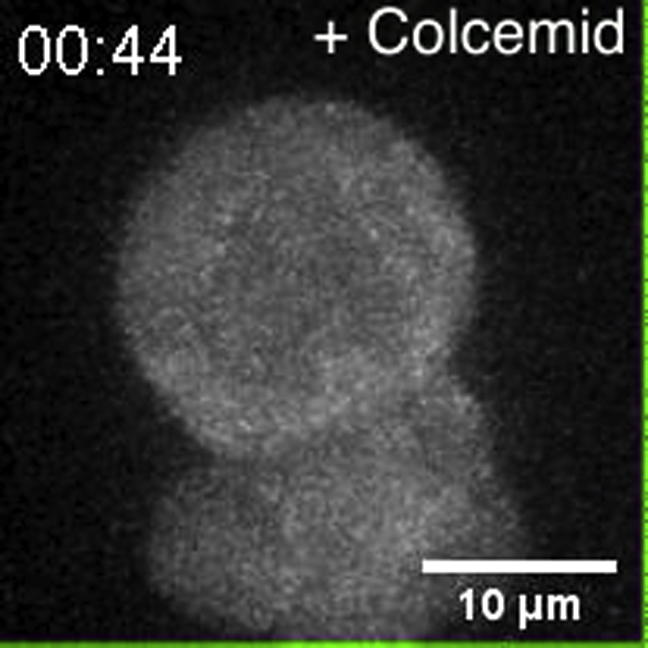

Supplement: Movie S5. MiraΔBH::mCherry Localizes to Cortical Microtubules in Interphase, Related to Figure 2 — A NB in primary cell culture expressing MiraΔBH::mCherry and treated with 50μM colcemid at the beginning of the movie. MiraΔBH::mCherry appears to localise to cortical microtubules since localized signal disappears upon colcemid treatment (15/19NBs). Time and scale as indicated. [file mmc6.jpg]
